# Erratum: PARP1 promotes gene expression at the post-transcriptional level by modulating the RNA-binding protein HuR

**DOI:** 10.1038/ncomms15191

**Published:** 2017-03-31

**Authors:** Yueshuang Ke, Yanlong Han, Xiaolan Guo, Jitao Wen, Ke Wang, Xue Jiang, Xue Tian, Xueqing Ba, Istvan Boldogh, Xianlu Zeng

Nature Communications 8 Article number:14632 ; DOI: 10.1038/ncomms14632 (2017); Published: 03 08 2017; Updated: 03 31 2017

The original version of this Article contained an error in the spelling of post-transcriptional in the title of the paper. This has now been corrected in both the PDF and HTML versions of the Article.

