# Supplementary material for: PARP1 promotes gene expression at the post-transcriptional level by modulating the RNA-binding protein HuR
Source: Nat Commun. 2017 Mar 8;8:14632. doi: 10.1038/ncomms14632 (PMC5344980; doi:10.1038/ncomms14632)
Supplement: Supplementary Information — Supplementary Figures [file ncomms14632-s1.pdf]

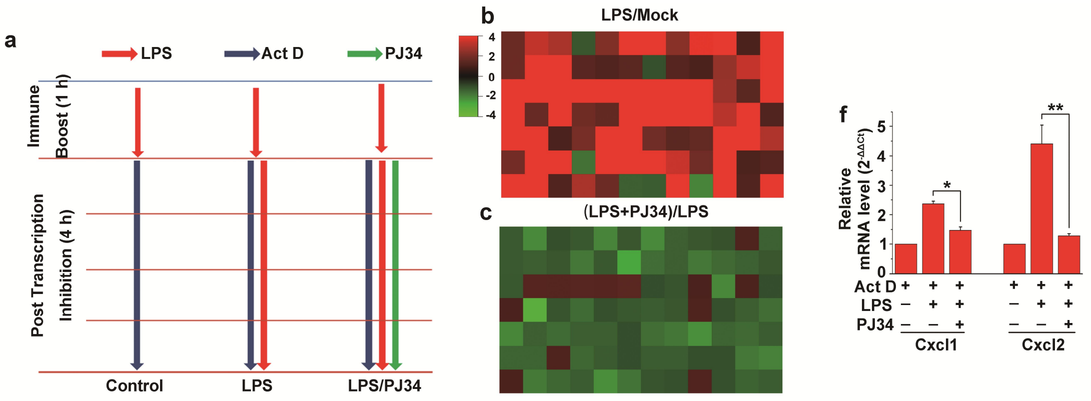

d

	1	2	3	4	5	6	7	8	9	10	11	12
A	Abcf1 1.72	Bcl6 3.31	Cxcr5 2.90	C3 -1.21	Casp1 2.20	Ccl1 7.77	Ccl11 14.92	Ccl12 2.33	Ccl17 3.85	Ccl19 16.18	Ccl2 1.05	Ccl20 4.48
B	Ccl22 1.74	Ccl24 10.60	Ccl25 5.63	Ccl3 1.36	Ccl4 1.16	Ccl5 1.36	Ccl6 -1.07	Ccl7 1.30	Ccl8 1.05	Ccl9 2.50	Ccr1 3.37	Ccr2 12.57
C	Ccr3 10.64	Ccr4 4.59	Ccr5 7.61	Ccr6 13.69	Ccr7 7.17	Ccr8 9.88	Ccr9 17.49	Crp 12.07	Cx3cl1 3.96	Cxcl1 2.60	Cxcl10 1.42	Cxcl11 9.33
D	Cxcl12 5.59	Cxcl13 1.64	Cxcl15 11.22	Pf4 1.84	Cxcl5 1.57	Cxcl9 4.40	Cxcr3 9.17	Ccr10 15.03	Ifng 11.76	Il10 3.11	Il10ra 4.94	Il10rb 1.18
E	Il11 13.45	Il13 5.13	Il13ra1 1.02	Il15 2.00	Il16 51.02	Il17b 8.89	Il18 1.85	Il1a 1.38	Il1b 1.59	Il1f6 8.89	Il1f8 15.58	Il1r1 2.99
F	Il1r2 1.93	Il20 25.18	Il2rb 9.38	Il2rg -1.71	Il3 15.70	Il4 9.50	Il5ra 10.75	Il6ra 3.40	Il6st 1.87	Cxcr2 7.99	Itgam 1.06	Itgb2 1.29
G	Lta 8.68	Ltb 11.43	Mif 1.19	Aimp1 2.86	Spp1 1.78	Tgfb1 -1.37	Tnf -1.33	Tnfrsf1a 5.61	Tnfrsf1b -2.07	Cd40lg 12.36	Tollip 1.18	Xcr1 17.43

e

	1	2	3	4	5	6	7	8	9	10	11	12
A	Abcf1 -1.27	Bcl6 -2.18	Cxcr5 -1.04	C3 -1.23	Casp1 -2.08	Ccl1 -1.21	Ccl11 -2.17	Ccl12 -1.38	Ccl17 -1.19	Ccl19 -1.11	Ccl2 1.13	Ccl20 -1.41
B	Ccl22 -1.28	Ccl24 -1.36	Ccl25 -1.20	Ccl3 -1.84	Ccl4 -1.23	Ccl5 -2.65	Ccl6 -1.63	Ccl7 -1.37	Ccl8 -1.83	Ccl9 -1.56	Ccr1 -1.41	Ccr2 -1.20
C	Ccr3 -1.22	Ccr4 1.28	Ccr5 1.26	Ccr6 1.30	Ccr7 1.28	Ccr8 1.39	Ccr9 -1.01	Crp -1.17	Cx3cl1 1.01	Cxcl1 -2.14	Cxcl10 1.09	Cxcl11 -1.02
D	Cxcl12 1.02	Cxcl13 -3.16	Cxcl15 -1.16	Pf4 -1.59	Cxcl5 -1.07	Cxcl9 -1.23	Cxcr3 -1.33	Ccr10 -1.19	Ifng 1.12	Il10 -1.10	Il10ra -1.36	Il10rb -1.65
E	Il11 -2.10	Il13 -1.37	Il13ra1 -1.58	Il15 -1.34	Il16 -1.36	Il17b -1.01	Il18 -1.67	Il1a -1.81	Il1b -2.29	Il1f6 -1.46	Il1f8 -1.57	Il1r1 -1.32
F	Il1r2 -1.46	Il20 -1.47	Il2rb 1.12	Il2rg -1.53	Il3 -1.29	Il4 -1.03	Il5ra -1.31	Il6ra -1.33	Il6st -1.05	Cxcr2 -1.03	Itgam -1.83	Itgb2 -2.03
G	Lta 1.05	Ltb -1.86	Mif -1.51	Aimp1 -1.31	Spp1 -2.27	Tgfb1 -1.74	Tnf -1.18	Tnfrsf1a -1.09	Tnfrsf1b 1.17	Cd40lg -1.07	Tollip -1.45	Xcr1 -1.31

Supplementary Figure 1 PARP1 is involved in regulating the stability of mRNAs from pro-inflammatory cytokine/chemokine mediators.

(a) A graphic depiction of the approach to determining the stability of mRNAs frompro-inflammatory mediators. Cells were subjected to immune boosts for 1 h and thentranscription was inhibited by the addition of Act D in the medium for up to 4 h. LPSand PARP inhibitor PJ34 were either present or not. RNA was extracted and real-timePCR was performed.

(b) Exposure to LPS increases the remaining mRNAs' levels of pro-inflammatory mediators of post-transcriptional inhibition.

(c) PARP1 inhibition decreases the remaining levels of pro-inflammatory

cytokine/chemokine mRNAs after transcription inhibition.

In (b) and (c), murine peritoneal macrophages (pMφ) were isolated from three mice, pooled and seeded in three 35-mm Petri dishes. Cells were exposed to LPS for 1 h and then subjected to transcriptional inhibition for 4 h in LPS and the PARP1 inhibitor PJ34 was either present, or not. The remaining mRNA levels were determined using Mouse Inflammatory Cytokines & Receptors q-PCR arrays. The heat maps show the fold changes in the mRNA levels of LPS-exposed cells compared with those of mock-treated cells (b) and the mRNA levels of LPS + PJ34-treated cells compared with just LPS-treated cells (c). Red, up-regulation; Green, down-regulation.

(d, e) Gene symbols in the PCR array plots. The positive or negative fold changes in mRNA levels corresponding to the magnitude of red or green color shown by the heat maps in b and c. Red, up-regulated (> 2-fold); green, down-regulated (> 2-fold).

(f) Remaining mRNA levels of *Cxcl1* (left panel) and *Cxcl2* (right panel). The cDNAs applied to PCR arrays were used to perform individual real-time PCR reactions to examine the *Cxcl1* and *Cxcl2* mRNA levels post-Act D addition in LPS-treated pMφ (±PJ34) using the primer pairs in the Materials and Methods. Data were expressed as mean ± SD (n=5), and analyzed by one-way ANOVA. * $p < 0.05$, ** $p < 0.01$.

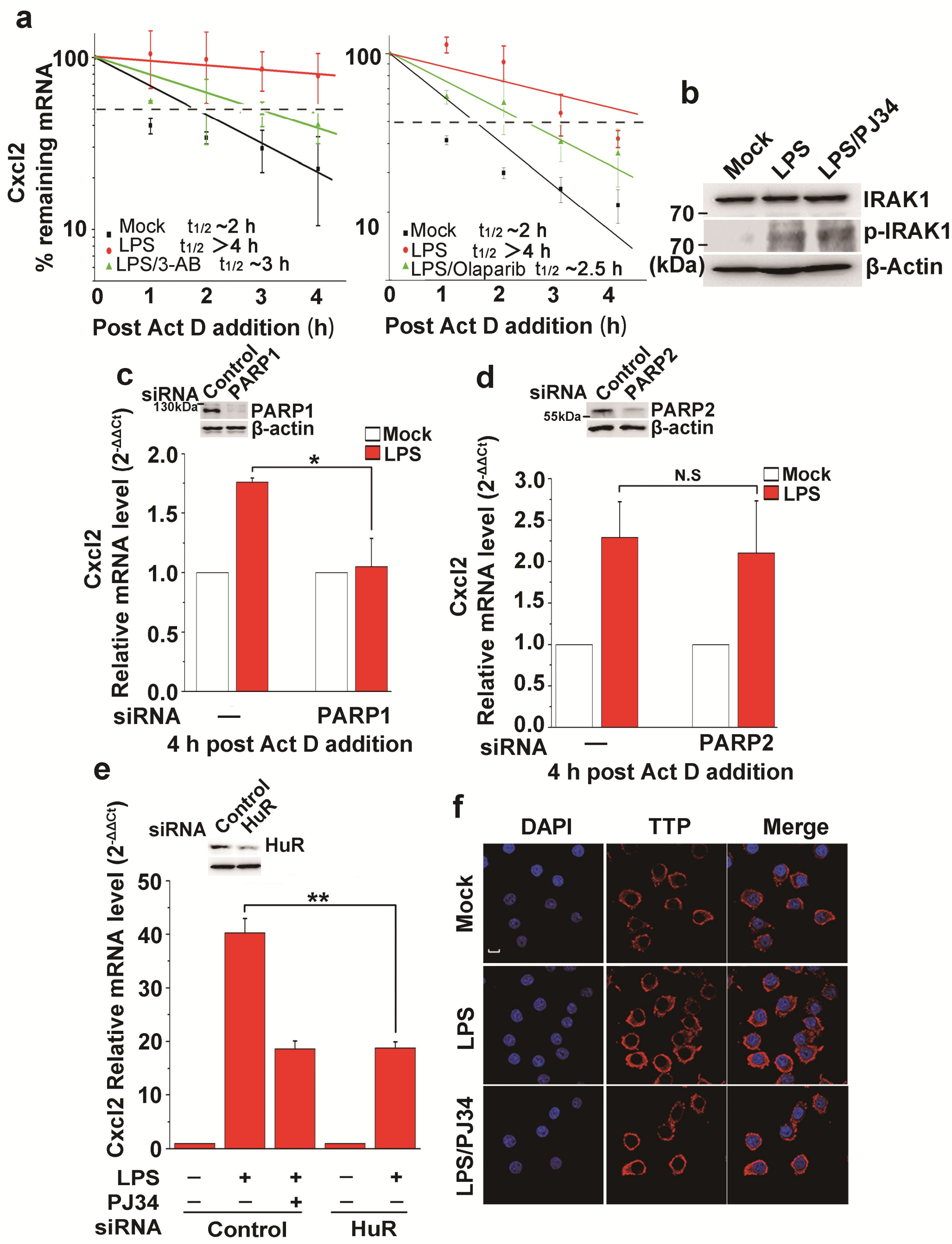

Supplementary Figure 2 PARP1 activation contributes to the HuR-mediated increase in stability of *Cxcl2* mRNA

(a) PARP1 activity is essential for the increase in *Cxcl2* mRNA half-lives in LPS-exposed macrophages. RAW 264.7 cells were exposed to LPS for 1 h and then subjected to transcriptional inhibition, with or without LPS maintenance, in presence or absence of 3-AB (left) or Olaparib (right) for different lengths of time (as indicated). Real-time PCR was performed to assess the remaining *Cxcl2* mRNA levels.

Half-lives of different samples are indicated in the insets.

(b) PARP1 inhibitor PJ34 does not impair TLR/inflammasome signaling. HEK 293 cells were LPS-exposed, with or without PJ34, for 2 h. Whole-cell lysates were prepared, and immunoblotting was performed to detect the levels of IRAK1 and p-IRAK1.

(c) Depletion of PARP1 decreases *Cxcl2* mRNA stability. The experiment was performed as described in the legend of Fig. 1C. A siRNA targeting PARP1 was utilized to measure *Cxcl2* mRNA stability.

(d) The depletion of PARP2 has no effect on *Cxcl2* mRNA stability. The experiment was performed as described in the legend of Fig. 1c. A small interfering RNA targeting PARP2 was utilized to measure *Cxcl2* mRNA stability.

(e) HuR is involved in the increase of *Cxcl2* mRNA induced by LPS stimulation. The experiment was performed as described in the legend Fig. 1g. Another siRNA targeting HuR was utilized to measure *Cxcl2* mRNA levels.

(f) The decay-promoting factor tristetraprolin is located in the cytoplasm of macrophages. RAW 264.7 cells were LPS-exposed, with or without PJ34, for 2 h. IF staining was conducted using a tristetraprolin antibody. TTP, tristetraprolin. Bar = 10 µm. Data were expressed as mean ± SD (n=5), and analyzed by one-way ANOVA. * $p < 0.05$, ** $p < 0.01$, NS, not significant.

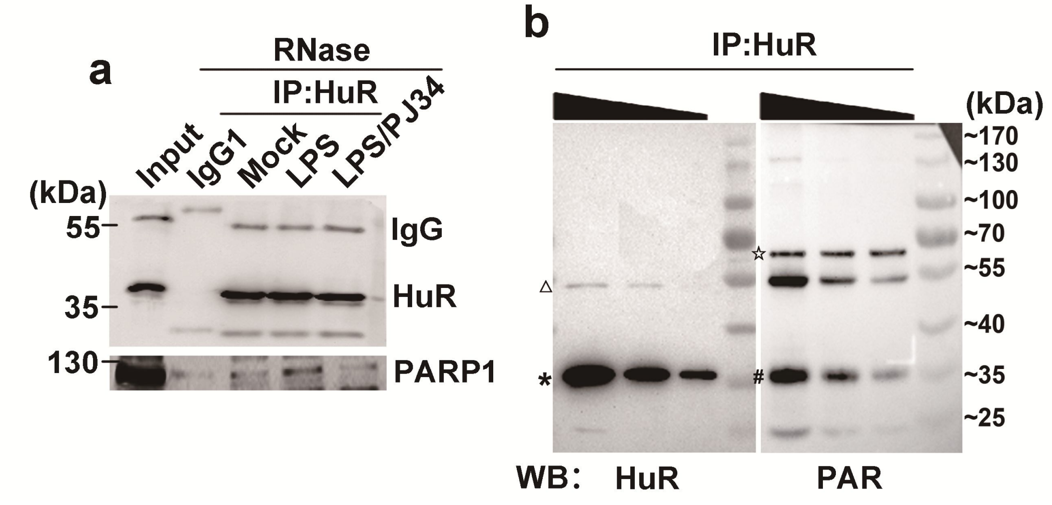

Supplementary Figure 3 PARP1 binds to, and PARylates, HuR.

(a) The binding of PARP1 with HuR does not require the existence of RNA. RAW 264.7 cells were LPS-exposed for 5 h. Whole cell extracts (WE) were prepared with the presence of Rnase (10 μg mL^{-1}). Immuno-precipitates were obtained using an antibody recognizing HuR. Immunoblotting was performed to detect levels of PARP1. (b) The PARylation of HuR in LPS-exposed cells. RAW 264.7 cells were LPS-exposed for 5 h. Whole-cell extracts were prepared and immuno-precipitates were obtained using an antibody recognizing HuR. Immunoblotting was performed to detect levels of HuR (left) and poly ADP-ribose (PAR) (right) in titrated samples. ☆, non-specific band. △, IgG. *, HuR. #, PAR.

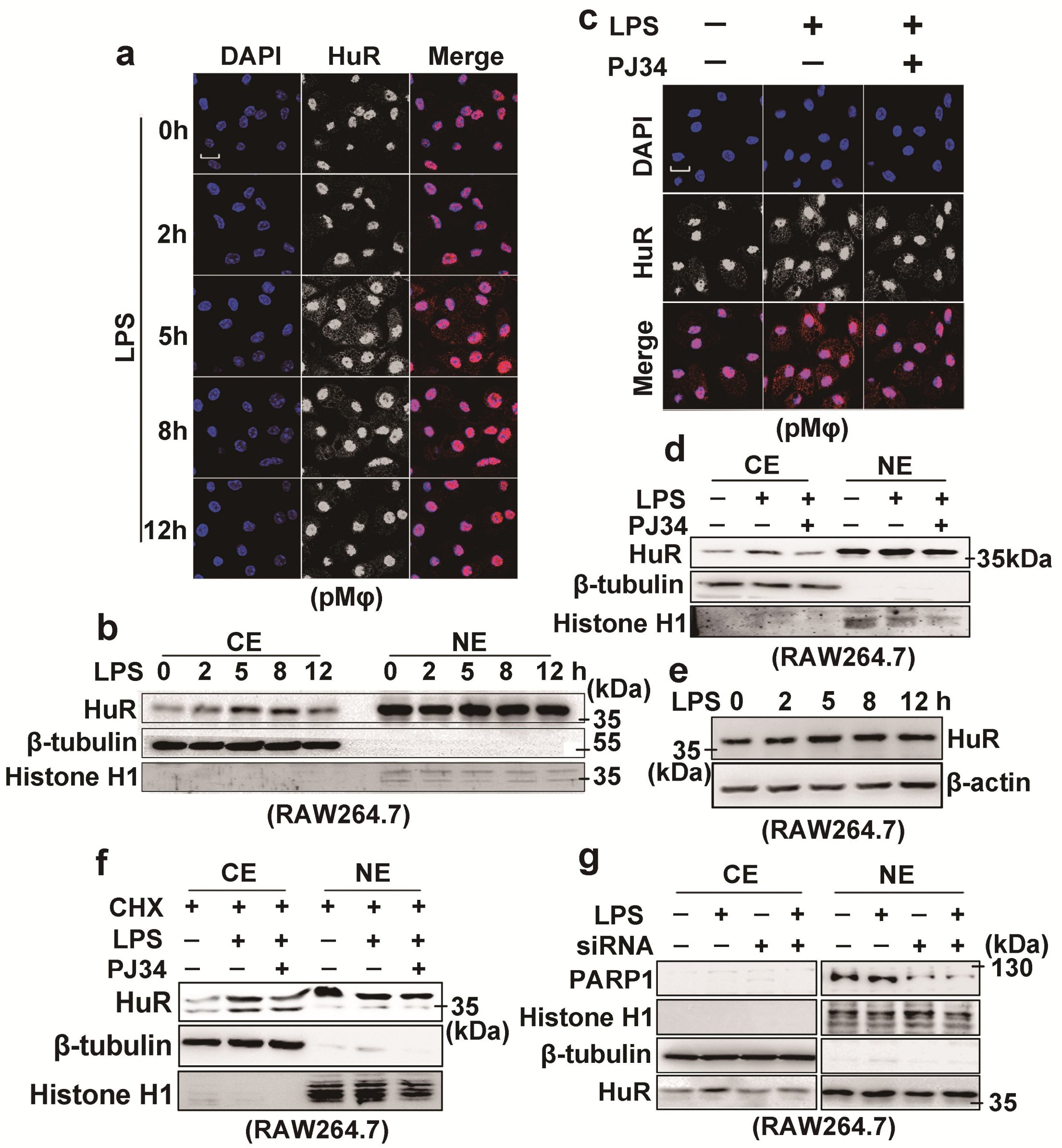

Supplementary Figure 4 PARP1 is involved in LPS-induced nuclear export of HuR.

(a, b) LPS stimulation induces the nuclear export of HuR. pMφ (a) and RAW264.7 cells were challenged with LPS for various lengths of time. The subcellular distribution of HuR is determined by IF staining. Bar = 10 μm (a), or cytosolic (CE) and nuclear (NE) extracts were prepared, and levels of HuR were determined by immunoblotting (b).

(c, d) PARP1 inhibition blocked HuR's nucleocytoplasmic shuttling induced by LPS. pMφ (c) and RAW264.7 cells were mock-challenged or LPS-exposed (± PJ34) for 5 h.

The subcellular distribution of HuR is determined by IF staining. Bar = 10 μm (c), or cytosolic (CE) and nuclear (NE) extracts were prepared, and levels of HuR were determined by immunoblotting (d).

(e) Time kinetics of HuR expression in LPS-exposed cells. RAW 264.7 cells were LPS-exposed for different times, as indicated. Whole-cell extracts were prepared, and immunoblotting was performed to detect HuR levels.

(f) The increased cytoplasmic level of HuR is not due to its enhanced protein synthesis. RAW 264.7 cells were exposed to LPS for 1 h and then subjected to protein synthesis inhibition by the addition of cycloheximide (CHX, 10 μg mL^{-1}). Cells withdrawn from LPS, maintained in LPS incubation, or treated with LPS + PJ34 were harvested, cytosolic (CE) and nuclear (NE) extracts were prepared, and levels of HuR were determined by immunoblotting,

(g) PARP1 silencing prevents HuR's nucleocytoplasmic shuttling induced by LPS. RAW 264.7 cells transfected with control or siRNA targeting PARP1 and then challenged with LPS for 5 h. Expression and distribution of PARP1 and HuR were examined by immunoblotting.

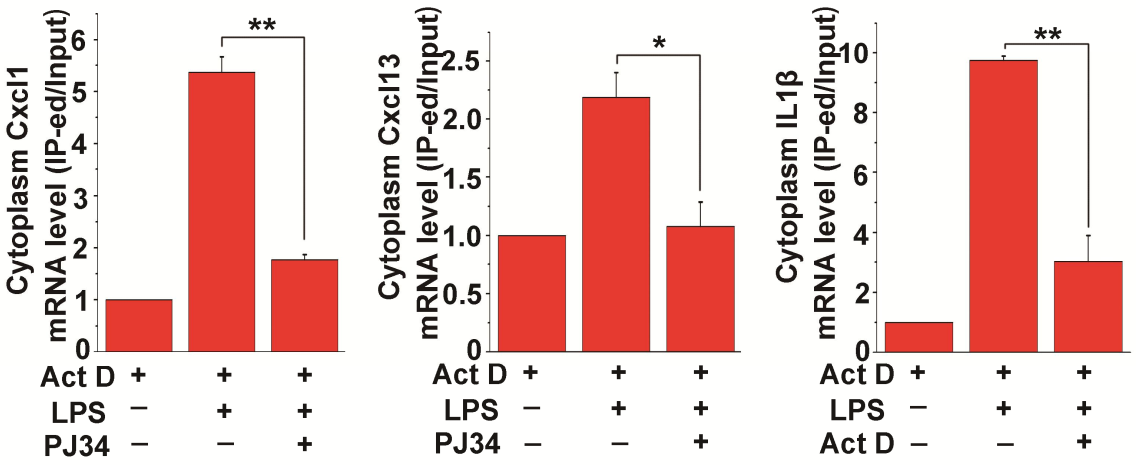

Supplementary Figure 5 PARP1 inhibition blocked the association of HuR with mRNA targets

PARylation promotes binding of HuR to ARE-containing mRNA. RAW 264.7 cells were exposed to 500 ng mL^{-1} LPS for 1 h to boost pro-inflammatory gene expression. After the addition of Act D, cells were withdrawn from LPS, maintained in LPS during incubation, or treated with LPS + PJ34 for another 2 h. Cytolic extracts (CEs) were prepared. RNA IP was conducted using a HuR antibody. Half of the bead–antibody–protein/mRNA complexes were utilized for immunoblotting to assess the equal loading/input of HuR, and the remaining half was subjected to real-time PCR to detect pulled-down *Cxcl1*, *Cxcl13* and *Il1β* mRNA levels using whole-cell lysate for calibration. Data were expressed as mean ± SD (n=5), and analyzed by one-way ANOVA. * $p < 0.05$, ** $p < 0.01$.

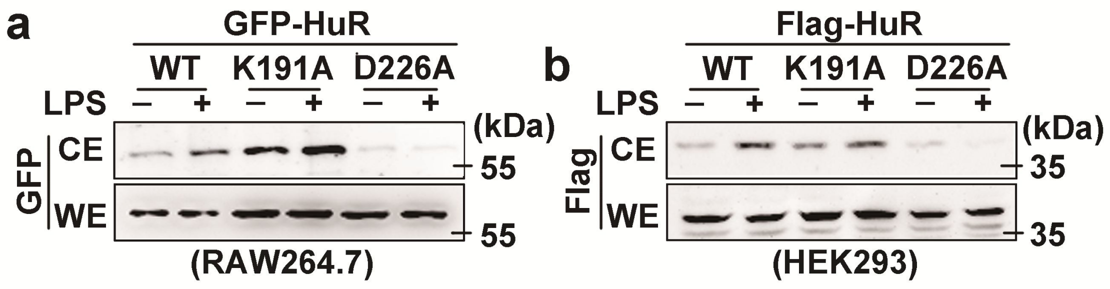

Supplementary Figure 6 The D226 mutant impacts nucleocytoplasmic shuttling of HuR.

(a) RAW264.7 cells transfected with wild-type (WT) GFP-HuR as well as K191A and D226A mutant plasmids, (b) HEK293 cells transfected with wild-type (WT) Flag-HuR as well as K191A and D226A mutant plasmids, and then challenged with or without LPS for 5 h. Cytosolic (CE) and whole cell (WE) extracts were prepared, and levels of HuR were determined by immunoblotting.

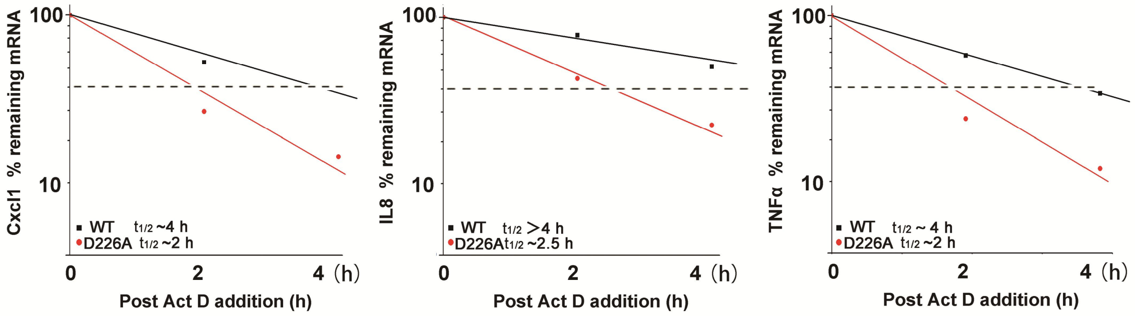

Supplementary Figure 7 Half-lives of ARE-containing mRNAs are decreased in D226A HuR-expressing cells in response to LPS exposure. Ectopic expression of murine WT HuR and D226A-HuR in endogenous HuR-silenced HEK 293 cells was carried out as described above in the legend to Figure 7g. Cells expressing WT or D226A HuR were stimulated with LPS for 1 h to boost inflammatory gene expression and were then subjected to transcriptional inhibition. Real time PCR was performed to assess the remaining *CXCL1*, *CXCL2* and *TNFα* mRNA levels. Half-lives of different samples are indicated in the inset.

Fig. 1

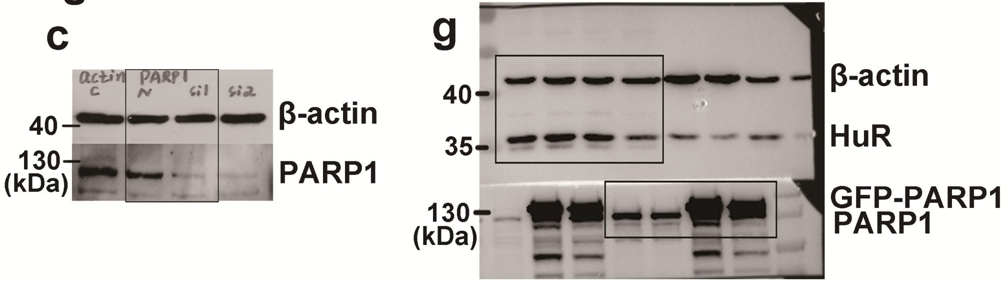

Fig. 2

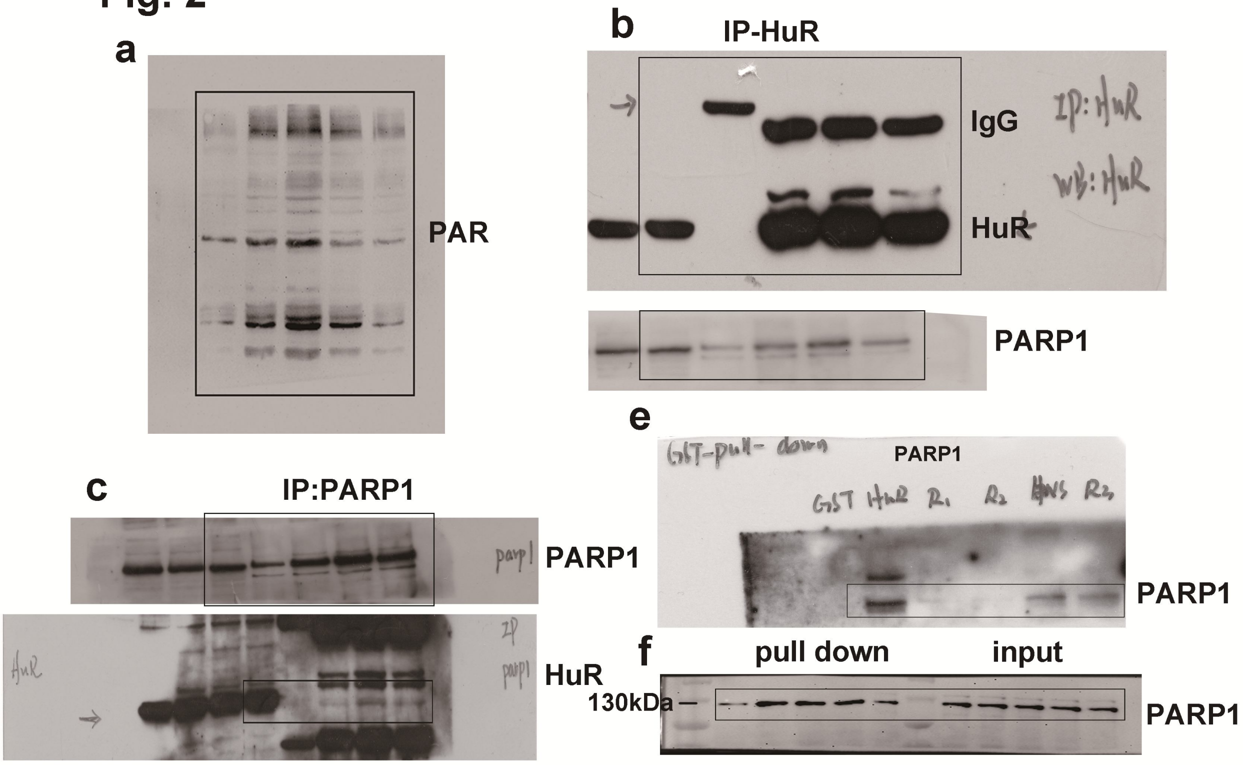

Supplementary Figure 8. Un-cropped scans of immunoblots.

Fig. 3

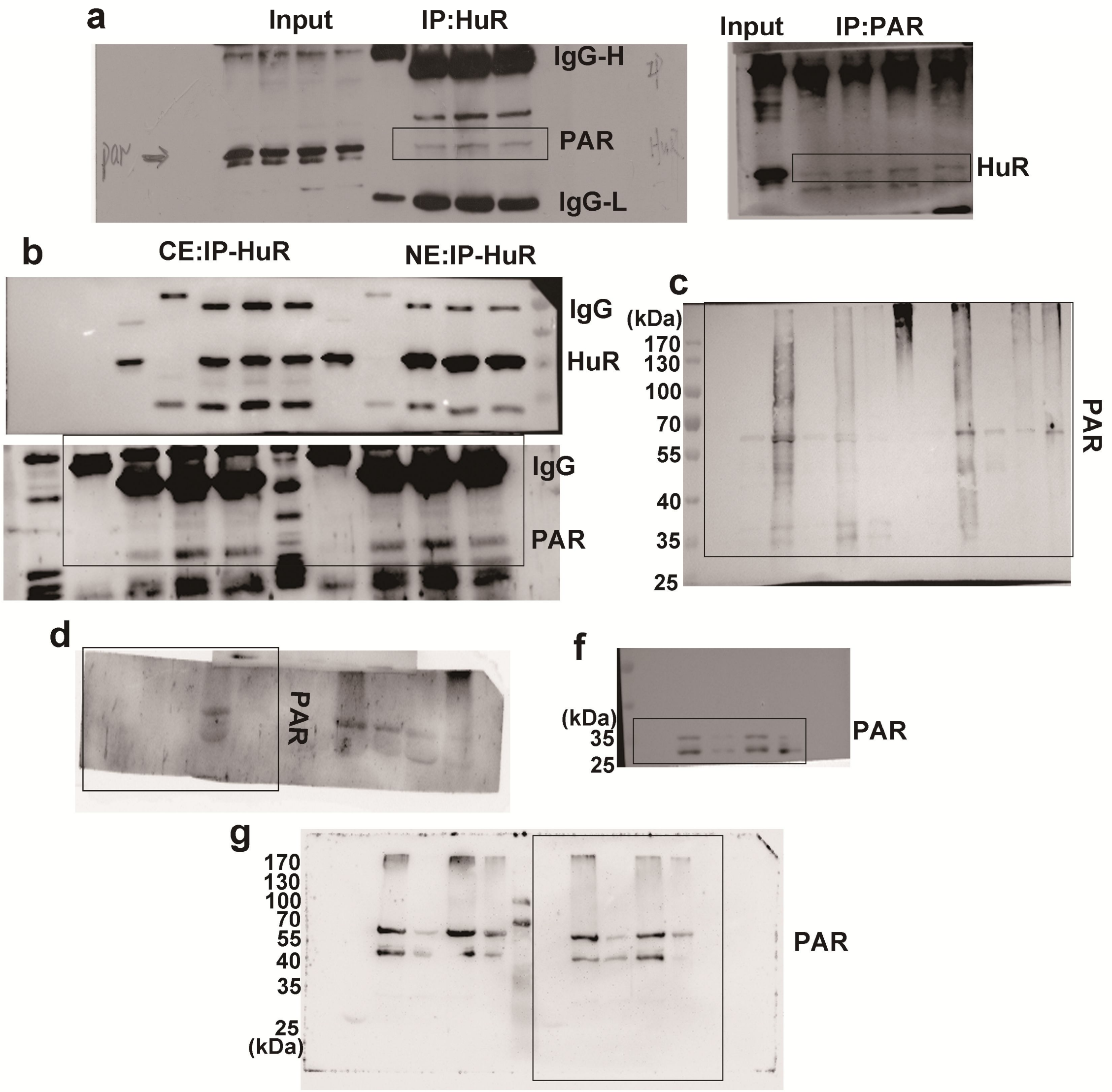

Supplementary Figure 8 continued.

Fig.8

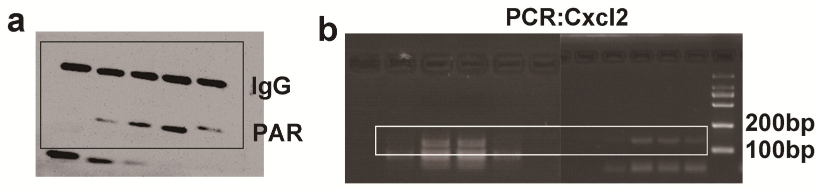

Fig.9

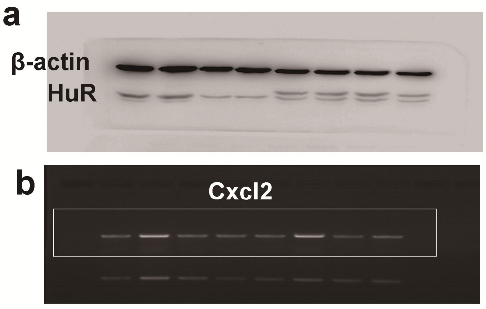

Fig.10

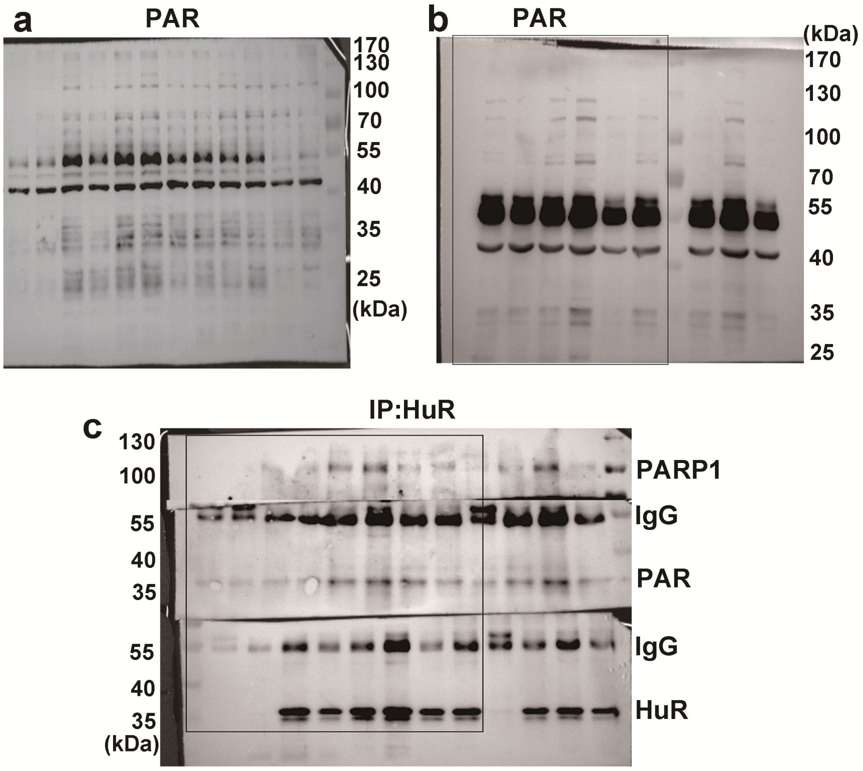

Supplementary Figure 8 continued.

Supplementary Figure 2d

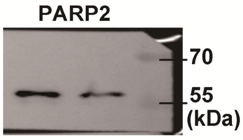

Supplementary Figure 4

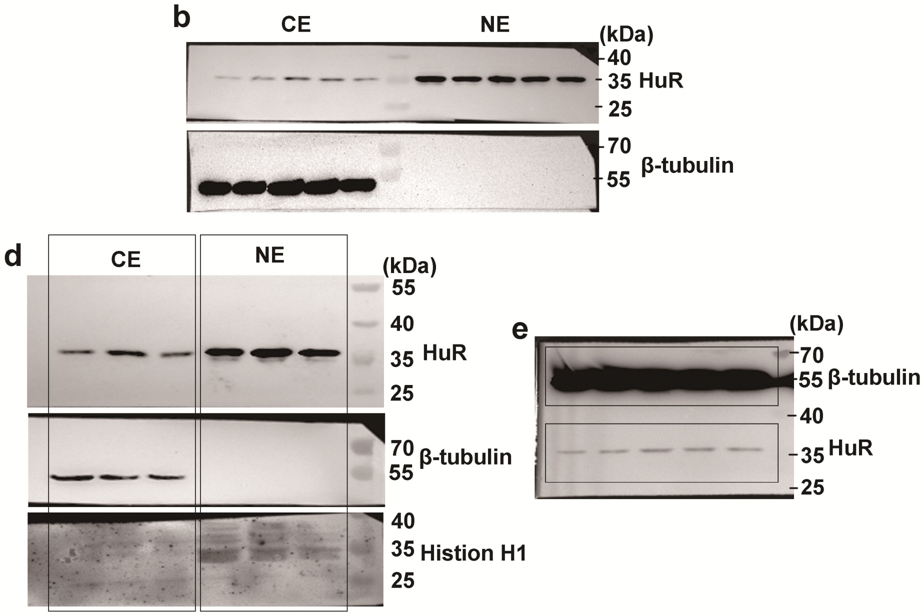

Supplementary Figure 6

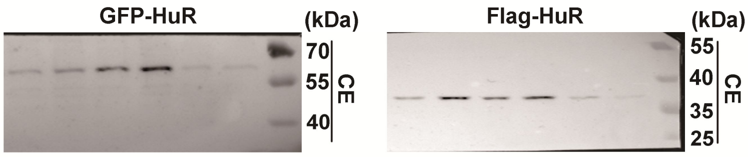

Supplementary Figure 8 continued. The un-cropped scans of some important blots shown in the main paper.
